# Metformin Attenuates Experimental Autoimmune Arthritis through Reciprocal Regulation of Th17/Treg Balance and Osteoclastogenesis

**DOI:** 10.1155/2014/973986

**Published:** 2014-08-20

**Authors:** Hye-Jin Son, Jennifer Lee, Seon-Yeong Lee, Eun-Kyung Kim, Min-Jung Park, Kyoung-Woon Kim, Sung-Hwan Park, Mi-La Cho

**Affiliations:** ^1^The Rheumatism Research Center, Catholic Research Institute of Medical Science, The Catholic University of Korea, 505 Banpo-dong, Seocho-gu, Seoul 137-701, Republic of Korea; ^2^Laboratory of Immune Network, Conversant Research Consortium in Immunologic Disease, College of Medicine, The Catholic University of Korea, 505 Banpo-dong, Seocho-gu, Seoul 137-701, Republic of Korea; ^3^Division of Rheumatology, Department of Internal Medicine, College of Medicine, The Catholic University of Korea, 505 Banpo-dong, Seocho-gu, Seoul 137-701, Republic of Korea; ^4^Division of Rheumatology, Department of Internal Medicine, Konkuk University School of Medicine, 120 Neungdong-ro, Gwangjin-gu, Seoul 143-701, Republic of Korea; ^5^Department of Life Science, College of Medicine, Laboratory of Immune Network, Rheumatism Research Center, Catholic Institutes of Medical Science, The Catholic University of Korea, 505 Banpo-dong, Seocho-gu, Seoul 137-701, Republic of Korea

## Abstract

Metformin is widely used to suppress certain functions of the cells found in diseases including diabetes and obesity. In this study, the effects of metformin on downregulating IL-17-producing T (Th17) cells, activating and upregulating regulatory T (Treg) cells, suppressing osteoclastogenesis, and clinically scoring collagen-induced arthritis (CIA) were investigated. To evaluate the effect of metformin on CIA, mice were orally fed with either metformin or saline as control three times a week for nine weeks. Histological analysis of the joints was performed using immunohistochemistry and Th17 cells and Treg cells of the spleen tissue were examined by confocal microscopy staining. Metformin mitigated the severity of CIA, reduced serum immunoglobulin concentrations, and reciprocally regulated Th17/Treg axis. Also, metformin treatment of normal cells cultured in Th17 conditions decreased the number of Th17 cells and increased the number of Treg cells. Metformin decreased gene expression and osteoclastogenic activity in CIA and normal mice. These results indicate that metformin had immunomodulatory actions influencing anti-inflammatory action on CIA through the inhibition of Th17 cell differentiation and the upregulation of Treg cell differentiation along with the suppression of osteoclast differentiation. Our results suggest that metformin may be a potential therapeutic for rheumatoid arthritis.

## 1. Introduction

Rheumatoid arthritis (RA) is a multisystem autoimmune disease of unknown etiology. The characteristic pathology of affected joints is the inflammation of hyperplastic synovial membrane that can result in destruction of adjacent cartilage and bone [1]. Though the exact molecular pathogenesis of RA remains elucidated, evidence suggests that interleukin- (IL-) 17-producing T cell, Th17, is a central player. IL-17 is known to act synergistically with tumor necrosis factor- (TNF-) *α* and IL-1*β*, which are abundant proinflammatory cytokines found in arthritic joints enhancing the activation of fibroblasts, chondrocytes, and osteoclasts [2]. In contrast, regulatory T (Treg) cell has been shown to have an anti-inflammatory role where its regulatory function is known to be deranged in RA patients. Th17 and Treg have been reported to have plasticity and can be converted to each other according to the cytokine milieu when the cells encounter [3]. Therefore, a therapeutic approach that can increase Treg cell while diminishing Th17 cell simultaneously may be very promising in RA treatment.

Metformin was originally introduced as an antidiabetic medication. Earlier studies have suggested that the majority of pharmacologic effect of metformin is dependent on its ability to activate AMP-activated protein kinase (AMPK), a major cellular regulator of lipid and glucose metabolism [4]. Metformin inhibits mitochondrial respiratory chain resulting in energy deficiency noted as an increase in AMP, activating AMPK and protein kinase A (PKA).

Activated AMPK and PKA inhibit lipid synthesis and gluconeogenesis, respectively [5]. AMPK activation also promotes glucose uptake and fatty acid oxidation in muscle, enhancing the lowering effect of glucose [6]. In addition to the glucose lowering effect, metformin has been reported to exert an anti-inflammatory effect, which is also mediated by metformin-activated AMPK. Metformin-activated AMPK suppresses mammalian target of rapamycin (mTOR), which regulates T cell effector differentiation* in vitro* and* in vivo*. AMPK has been shown to be associated with Th17 cell suppression by inhibiting mTOR and signal transducer and activator of transcription 3 (STAT3), suggesting the therapeutic potential of AMPK agonist [7].

While Th17 cells are responsible for perpetuating chronic inflammation in RA, osteoclasts resorb bone resulting in subsequent joint destruction [8]. The role of mTOR in osteoclastogenesis has been reported where a downstream molecule of the mTOR pathway (S6K) conveys cell survival signal in osteoclasts [9]. Recently, Indo et al. reported that AMPK-mediated inhibition of mTOR and hypoxia-induced factor (HIF)-1*α* negatively regulated osteoclastogenesis [10].

These regulatory effects of AMPK on Th17 cells and osteoclasts have prompted the investigation on the effect of the AMPK agonist, metformin, on autoimmune arthritis. In the present study, the suppression of metformin in a collagen-induced arthritis (CIA) mouse model was demonstrated by focusing on the suppression of Th17 differentiation and enhancing Treg differentiation. Phosphorylation of STAT3 was diminished by metformin while phosphorylation of STAT5 increased, which seems to contribute to the alteration of Th17/Treg population. In addition, osteoclastogenesis was suppressed via inhibition of mTOR and AMPK activation by metformin.

## 2. Materials and Methods

### 2.1. Mice

12-week-old male C57BL/6 mice (Orient Bio, Korea) were maintained under specific pathogen-free conditions and fed standard laboratory mouse chow (Ralston Purina, St. Louis, MO) and water* ad libitum*. All experimental procedures were examined and approved by the Animal Research Ethics Committee of the Catholic University of Korea, which conforms to all National Institutes of Health of the USA guidelines. All surgeries were performed under isoflurane anesthesia and all efforts were made to minimize suffering.

### 2.2. Induction of Arthritis and Treatment of Metformin

CIA was induced in C57BL/6 mice (*n* = 20). Mice were immunized into the base of the tail with 100 *μ*g of chicken CII (Chondrex Inc., Redmond, WA, USA) in complete Freund's adjuvant (Chondrex Inc.). 100 *μ*g of chicken CII in incomplete Freund's adjuvant (Chondrex Inc.) was injected at tail and one foot on day 14. CIA mice were orally fed 3 times a week for 9 weeks with 5 mg/mouse metformin (Sigma-Aldrich) or saline as a control beginning on day 7 after first immunization (permit number: CUMC-2013-0128-01). Arthritis in these mice was examined visually two times per week for the appearance of arthritis in the peripheral joints.

### 2.3. Clinical Scoring of Arthritis

The severity of arthritis was recorded using the mean arthritis index on scale of 0–4, as previously reported [11], as follows: (0), no evidence of erythema and swelling; (1), erythema and mild swelling confined to the midfoot (tarsals) or ankle joint; (2), erythema and mild swelling extending from the ankle to the midfoot; (3), erythema and moderate swelling extending from the ankle to the metatarsal joints; (4), erythema and severe swelling encompassing the ankle, foot, and digits. The severity of arthritis was analyzed by the sum of scores from all legs, assessed by two independent observers with no knowledge of the experimental groups.

### 2.4. Immunohistopathological Analysis of Arthritis

Joints of each mouse were fixed in 10% formalin, decalcified in 10% EDTA, and embedded in paraffin wax. The sections were stained with hematoxylin-eosin (H&E), safranin O, and toluidine blue to detect proteoglycans. For immunohistochemistry, the sections were performed using the Vectastain ABC kit (Vector Laboratories, Burlingame, CA, USA) [12]. Inflammation was scored according to criteria previously reported. [13]. Tissues were stained anti-receptor activator of NF-*κ*B (RANK), anti-receptor activator of NF-*κ*B ligand (RANKL), anti-TNF-*α*, anti-TNF receptor-associated factor 6 (TRAF6), anti-IL-17, and anti-IL-6 (all from Santa Cruz Biotechnology Inc.) and anti-phosphorylated(p) STAT3 (Y705), anti-pSTAT3 (S727), and anti-pAMPK anti-pmTOR (all from Cell Signaling, Danvers, MA).

### 2.5. Confocal Microscopy of Immunostaining

Spleen tissues were obtained on day 35 after first immunization. The tissue was stained using PE-conjugated anti-CD4, FITC-conjugated anti-forkhead box P3 (Foxp3), APC-conjugated anti-CD25, FITC-conjugated anti-IL-17, FITC-conjugated anti-pSTAT3 (Y705), and FITC-conjugated anti-pSTAT3 (S727) (all from eBiosciences, San Diego, CA, USA). Stained sections were analyzed using a Zeiss microscope (LSM 510 Meta; Carl Zeiss, Oberkochen, Germany).

### 2.6. Measurement of Immunoglobulin (Ig) Concentrations

Anti-IgG, IgG1, and IgG2a were measured by mouse IgG, IgG1, and IgG2a ELISA quantitation kits (Bethyl Laboratories, Montgomery, TX) [12].

### 2.7. Flow Cytometry of Intracellular Cytokines

To analyze intracellular cytokines, splenocytes were stained with PerCP-conjugated anti-CD4, APC-conjugated anti-CD25, FITC-conjugated anti-IL-17, and PE-conjugated anti-Foxp3 (eBiosciences), followed by fixation and permeabilization with a Foxp3 staining buffer kit (BD Bioscience) according to the manufacturer's instructions. Four hours before the staining, the cells were stimulated with phorbol myristate acetate (25 ng/mL) and ionomycin (250 ng/mL) (all from Sigma-Aldrich) and GolgiStop (BD Bioscience). All data was analyzed using FlowJo software (Tree Star, Ashland, OR, USA).

### 2.8. Real-Time Quantitative Polymerase Chain Reaction (PCR)

The mRNA expression levels were estimated using a LightCycler 2.0 instrument (Roche Diagnostic, Mannheim, Germany) with the version 4.0 software. All reactions were performed with the LightCycler FastStart DNA Master SYBR Green I (Takara, Shiga, Japan), following the manufacturer's instructions. The mRNA expression was normalized to that of *β*-actin. The primers sequences are shown in Table 1.

### 2.9. *In Vitro* Differentiation into Th17 Cells

To purify splenic CD4+ T cells, the splenocytes were incubated with CD4-coated magnetic beads and isolated using magnetic activated cell sorting (MACS) separation columns (Miltenyi Biotec). Isolated CD4+ T cells were stimulated with Th17 cell-polarizing conditions for 3 days: plate-bound anti-CD3 (0.5 *μ*g/mL), anti-CD28 (1 *μ*g/mL) (both from BD PharMingen, CA, USA); anti-interferon (IFN)-*γ* (2 *μ*g/mL), anti-IL-4 (2 *μ*g/mL), transforming growth factor (TGF)-*β* (2 ng/mL), and IL-6 (20 ng/mL) (all from R&D Systems, Minneapolis, MN, USA).

### 2.10. *In Vitro* Osteoclastogenesis

Osteoclasts were stimulated in the presence of macrophage colony-stimulating factor (M-CSF) (10 ng/mL) (R&D Systems) and receptor activator of nuclear factor kappa-B ligand (RANKL) (50 ng/mL) (PeproTech, London, UK) and absence or presence of metformin 1 mM. The medium was changed every two days. Osteoclasts were generated after 8–10 days [14].

### 2.11. Western Blot Analysis

The protein was separated by SDS-PAGE and transferred on nitrocellulose membranes (Amersham Pharmacia Biotech, Piscataway, NJ, USA). Western blot was performed by SNAP i.d. protein detection system (Millipore). The hybridized bands were detected by enhanced chemiluminescence (ECL) detection kit (Thermo Scientific brand of Thermo Fisher Scientific, Inc.). The antibodies were as follows: anti-AMPK, anti-*p*AMPK, anti-mTOR, anti-*p*mTOR, anti-*p*STAT3 Y705, anti-*p*STAT3 S727, and anti-STAT3 (all from Cell Signaling); TRAF6 (Santa Cruz); and *β*-actin (Sigma).

### 2.12. Statistical Analysis

All data were expressed as the mean ± SD. Statistical analysis was performed using SPSS 10.0 for Windows (IBM Corp., Armonk, NY). Comparing numerical data between groups was performed with 2-way ANOVA and nonparametric Mann-Whitney tests. Differences in the mean values of various groups were analyzed by using ANOVA with a post hoc test.* P* values < 0.05 were considered significant.

## 3. Results

### 3.1. Metformin Ameliorates Collagen-Induced Arthritis

**Figure 1:**
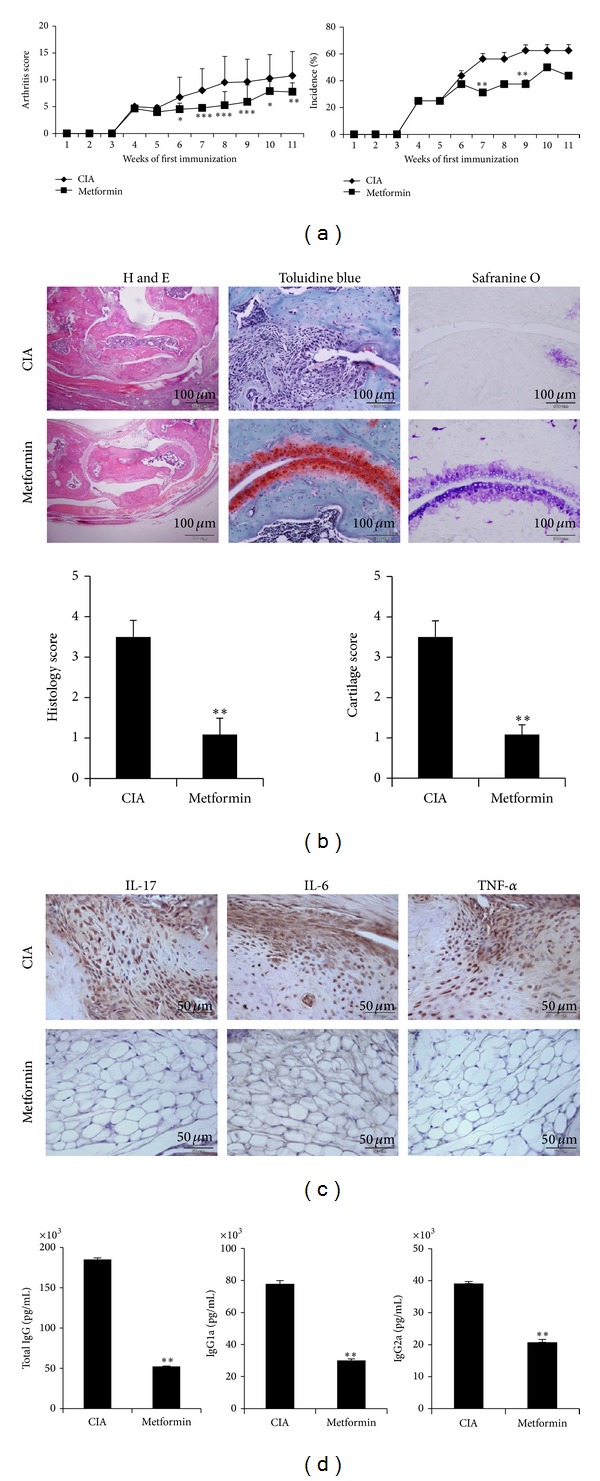
Therapeutic effects of metformin in CIA model. CIA was induced in C57BL/6 mice. Metformin 5 mg/mice (*n* = 10) or saline (*n* = 10) was oral feed three times into CIA in a week. Mice were sacrificed on day 70 after first immunization. (a) Clinical arthritis scores were determined. (b) The joint tissues from CIA: metformin-treated CIA mice were stained with H&E, safranin O, and toluidine blue (original magnification, ×200). The average histopathological score is shown in bar graphs (below) (scale bar = 100 *μ*m). (c) Immunohistochemical detection of IL-17, IL-6, and TNF-*α* was stained in the synovium of CIA and metformin-treated CIA. All tissues were counterstained with hematoxylin (original magnification, ×400). All images were obtained for each mouse (*n* = 10), showing representative images (scale bar = 50 *μ*m). (d) Mice serum was obtained on day 30 after CII immunization. The serum obtained after first immunization. The levels of IgG, IgG1, and IgG2a antibodies were measured from each group. Mean ± SD of three independent experiments (*P < 0.05; **P < 0.01).

To investigate the antiarthritic effect of metformin, mice with CIA were orally fed with either metformin or saline three times a week from day 7 after first immunization. Metformin-treated CIA mice showed significantly reduced severity (Figure 1(a), left) and incidence of clinical arthritis (Figure 1(a), right). Histological and cartilage scores measured based on inflammatory cell infiltration and cartilage damage were also significantly lower in metformin-treated mice (Figure 1(b)). IL-17, IL-6, and TNF positive cells decreased in metformin-treated CIA mice (Figure 1(c)). Total IgG, IgG1, and IgG2a antibodies significantly decreased in metformin-treated CIA mice when compared to saline-treated controls (Figure 1(d)). Collectively, these results demonstrated that metformin attenuated CIA.

### 3.2. Analysis of the Th17, Treg Cell Population in CIA Mice

**Figure 2:**
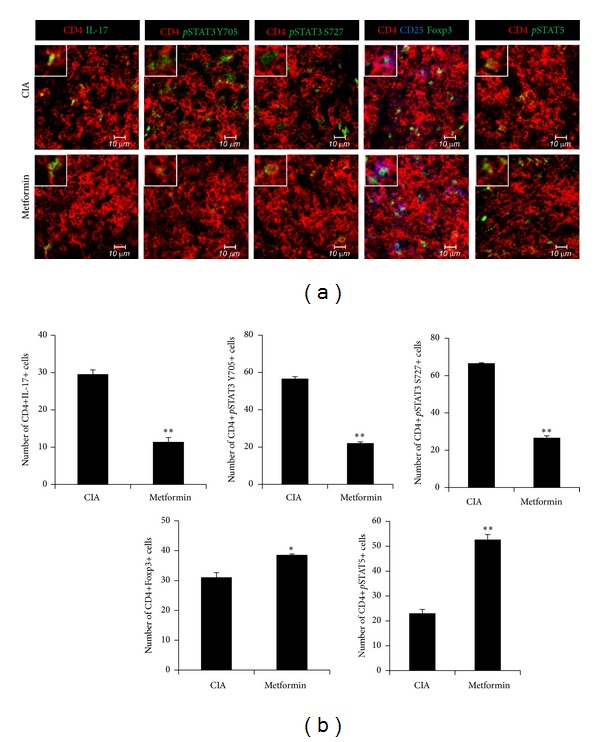
Regulation of Th17 cells and Foxp3+ regulatory T cells in CIA spleen. Spleen tissue was obtained from metformin-treated CIA and control CIA on day 35 after first immunization. (a) Confocal staining examined by antibodies: Th17 cell was stained with CD4 (red) and IL-17 (green). CD4+ CD25+ Foxp3+ regulatory T cells were stained with CD4 (red), CD25 (blue), and Foxp3 (green). For activated STATs analysis, the tissues were stained with CD4 and* p*STAT3 S727,* p*STAT3 Y705, or* p*STAT5. All images were performed for each mouse (*n* = 5), showing representative images (scale bar = 10 *μ*m). (b) The mean values are presented in the form of a histogram by four individuals. Results are shown as mean ± SD (*n* = 5 mice per group). Mean ± SD of three independent experiments (*P < 0.05; **P < 0.01).

**Figure 3:**
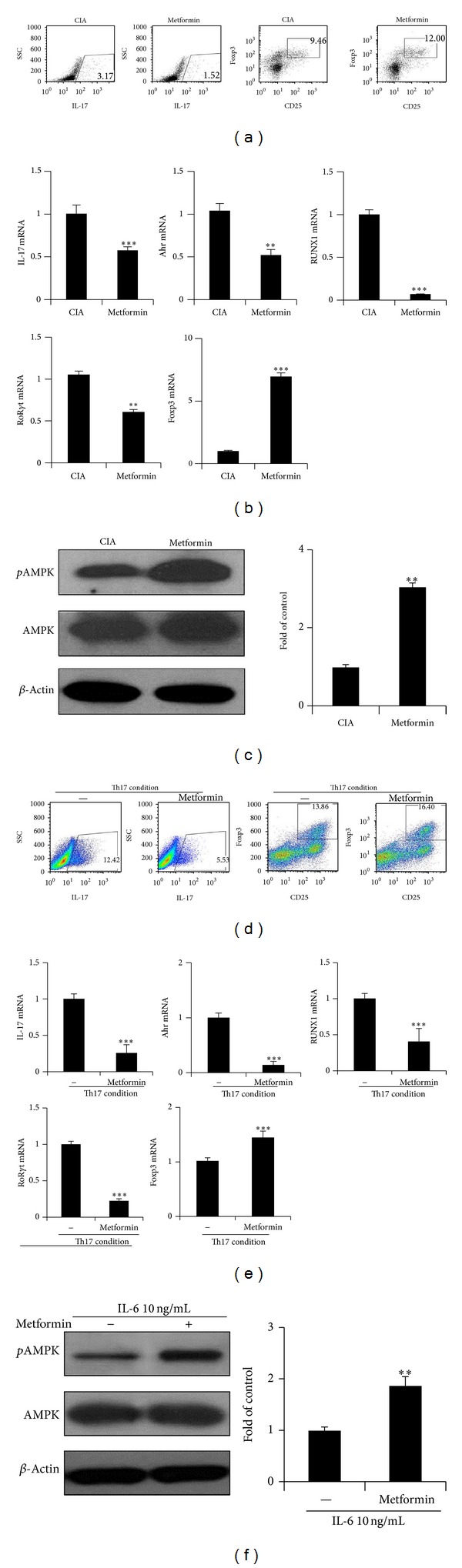
Treatment with metformin Treg cells and decreases Th17 cells in CIA mice and* in vitro* Th17 polarizing condition. (a–c) Splenocytes were also obtained from metformin-treated CIA (*n* = 5) and control CIA (*n* = 5) on day 35 after first immunization. (a) Both isolated CD4+ T cells were stained with anti-CD25, anti-Foxp3, and anti-IL-17 antibody. The proportions of CD4+IL-17+ T cells and CD4+CD25+ Foxp3+ regulatory T cells were analyzed using flow cytometry. (b) The gene levels of IL-17, Ahr, RUNX1, ROR*γ*T, and Foxp3 in splenocytes were determined by real-time PCR. (c) The expressions of phosphorylated AMPK were measured by western blot. The fold of control measured* p*AMPK/AMPK/*β*-actin ratio (right). (d–f) Isolated CD4+ T cells of C57BL/6 mice were cultured with under Th17 polarizing conditions in the presence or absence of 1 mM metformin for 3 days. (d) The cells were stained with anti-CD4, anti-CD25, anti-IL-17, and anti-Foxp3. (e) The mRNA expression levels of IL-17, Ahr, RUNX1, ROR*γ*T, and Foxp3 were determined by real-time PCR. (f) CD4+ T cells were stimulated with IL-6 10 ng/mL in the presence or absence of metformin 1 mM for 1 hour. The activation of* p*AMPK was measured by western blot. The representative results are shown in the right panel. Data are presented as the mean ± SD of four independent experiments (*P < 0.05; **P < 0.01; ***P < 0.005).

We investigated the effect of metformin on Th17, Treg population in CIA mice. First, we was analyzed for Th17 and Treg cells using tissue confocal staining. The numbers of CD4+IL-17+ T cells and CD4+* p*STAT3+ (both at S727 and Y705) T cells were reduced in metformin-treated CIA mice more than in the controls. In contrast, the numbers of CD4+ CD25+ Foxp3+ Treg cells and CD4+* p*STAT5+ T cells were higher in the metformin-treated group (Figure 2). Next, spleen cells isolated from each group were analyzed for the expression of IL-17 and Foxp3 using flow cytometry. The results showed that CD4+IL-17+Th17 cells were lower in the metformin-treated CIA, whereas CD4+CD25+ Foxp3+Treg cells were increased in metformin group (Figure 3(a)). As well, mRNA expressions of the Th17-related cytokines and transcription factors were significantly reduced in the splenocytes from metformin-treated CIA mice and there was greater expression of Foxp3 (Figure 3(b)). The* p*AMPK expression increased in metformin-treated CIA (Figure 3(c)).

### 3.3. Metformin Regulates Th17, Treg Cell Differentiation* In Vitro*

As the proportion of Th17 cell was decreased in metformin-treated CIA mice* in vivo*, the effect of metformin on Th17 cell and Treg cell differentiation* in vitro* was examined. CD4+ T cells isolated from healthy C57BL/6 mice were cultured in Th17 cell-polarizing condition in the presence or absence of metformin. The differentiation of Th17 cells was inhibited with metformin as there was a simultaneous increase in Treg differentiation (Figure 3(d)). In addition, metformin inhibited the expression levels of the Th17-associated genes, IL-17, Ahr, RUNX1, and ROR*γ*T, as it increased mRNA levels of Foxp3 (Figure 3(e)). Treatment with metformin increased the expression of* p*AMPK in IL-6 10 ng/mL stimulated CD4 T cell (Figure 3(f))). These results indicate that metformin suppresses Th17 cell differentiation while enhancing Treg differentiation and AMPK activation in a proinflammatory condition (Figure 6).

### 3.4. The Effect of Metformin on Osteoclastogenesis* In Vivo*

**Figure 4:**
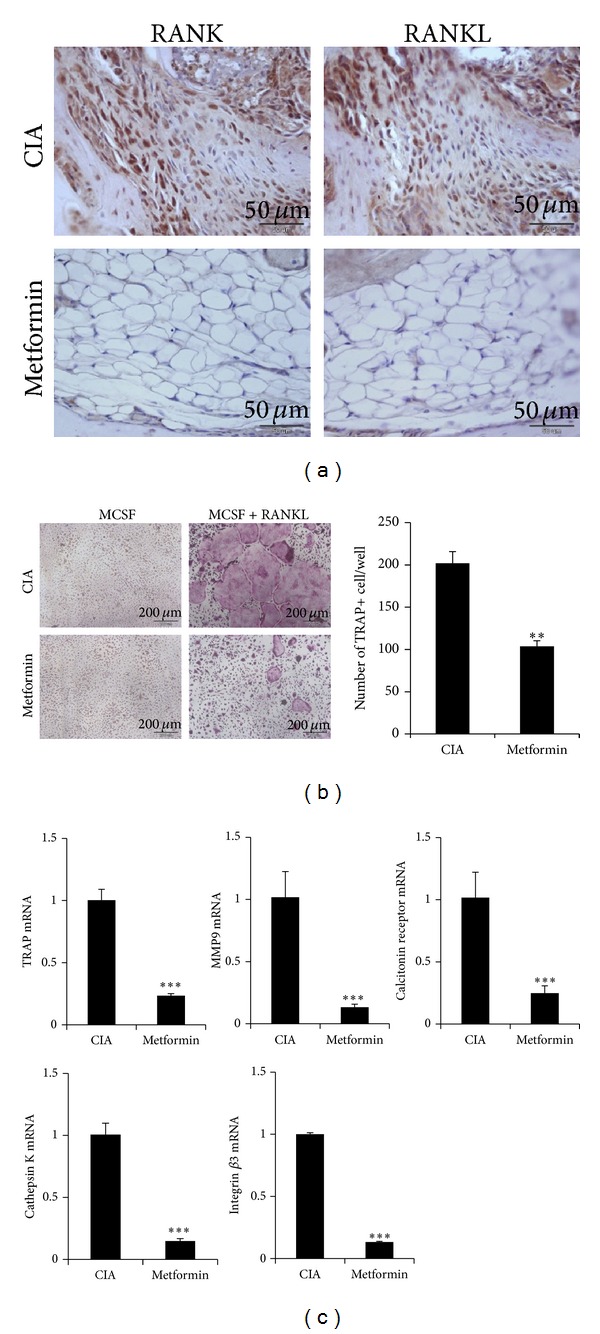
Metformin inhibits osteoclast formation in CIA mice. (a) Joint tissues of CIA control mice (*n* = 5) and metformin-treated CIA (*n* = 5) were stained with anti-RANKL and anti-RANK antibodies 70 days after the first immunization. All images were obtained for each mouse (*n* = 5), showing representative images (scale bar = 50 *μ*m). (b) Isolated preosteoclasts from each group were cultured with 10 ng/mL M-CSF and/or 50 ng/mL RANK. Differentiated osteoclasts were stained with TRAP. The TRAP+ cells were indicated in graph (right) (scale bar = 200 *μ*m). (c) The mRNA expressions of TRAP, MMP-9, calcitonin receptor, cathepsin K, and integrin *β*3 as osteoclast markers were quantified by real-time PCR. Data are presented as the mean ± SD of four independent experiments (*P < 0.05; **P < 0.01; ***P < 0.005).

Metformin-treated CIA mice showed reduced bone erosion and cartilage destruction in the arthritic joints (Figure 1(b)). These results have prompted us to investigate direct effect of metformin on osteoclastogenesis. To determine effect of metformin on osteoclastogenesis, we were immunochemical staining for RANK and RANKL in joint tissues. As expected, the expression of RANK and RANKL decreased in the inflamed joint tissue of metformin-treated CIA (Figure 4(a)). The preosteoclasts derived from the control CIA mice and metformin-treated CIA mice were stimulated with M-CSF and RANKL. The results showed that the osteoclasts from metformin-treated CIA mice were less capable of differentiating into osteoclasts (Figure 4(b)). The expression levels of various osteoclastogenic markers, such as TRAP, MMP-9, integrin *β*3, calcitonin receptor, and cathepsin K, were decreased, also. The reduction in osteoclastic activity was observed following metformin-treated mice (Figure 4(c)).

### 3.5. Metformin Suppresses Osteoclast Differentiation by Inhibiting the STAT3, AMPK Pathway

**Figure 5:**
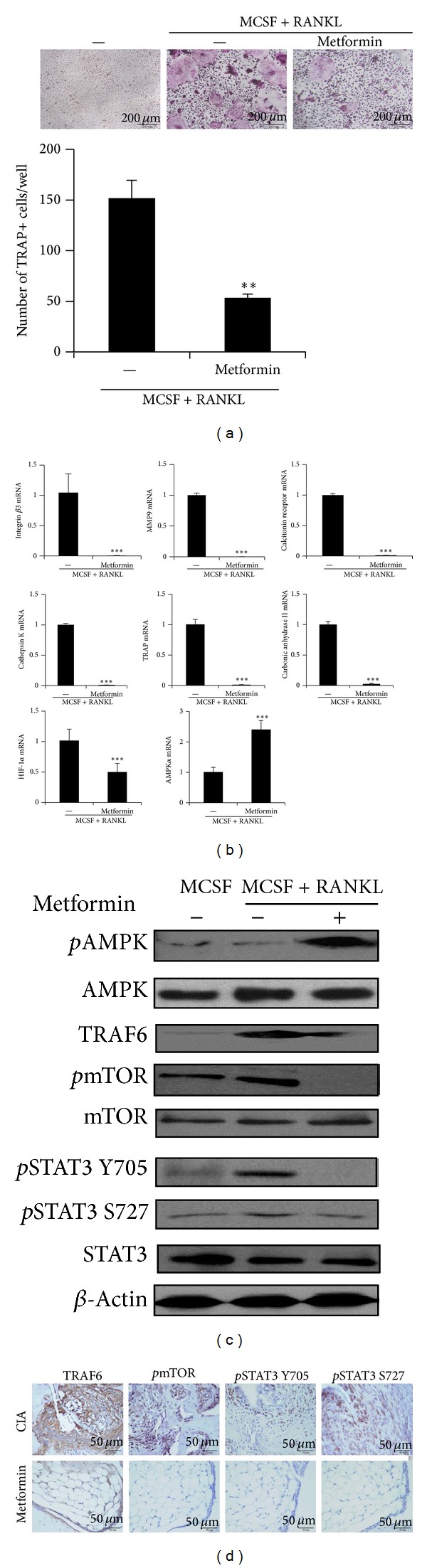
Metformin suppresses osteoclastogenesis* in vitro*. C57BL/6 mice preosteoclasts were cultured in the presence of 10 ng/mL M-CSF and/or 50 ng/mL RANKL in the presence or absence of metformin 1 mM. (a) Differentiated osteoclasts were stained for TRAP (scale bar = 200 *μ*m) and The TRAP+ cells were indicated in graph (under). (b) The mRNA levels of TRAP, MMP-9, calcitonin receptor, carbonic anhydrase II, cathepsin K, integrin *β*3, HIF1-*α*, and AMPK were quantified by real-time PCR. (c) Protein levels of* p*AMPK, TRAF6,* p*mTOR, mTOR,* p*STAT3 Y705,* p*STAT3 S727, STAT3, and *β*-actin were analyzed using Western blot in osteoclasts. (d) Immunohistochemical detection of TRAF6,* p*mTOR* p*STAT3 Y705, and* p*STAT3 S727 were stained in the synovium of CIA and metformin-treated CIA. All tissues were counterstained with hematoxylin (original magnification, ×400) (scale bar = 50 *μ*m). Data are presented as the mean ± SD of four independent experiments (*P < 0.05; **P < 0.01; ***P < 0.005).

**Figure 6:**
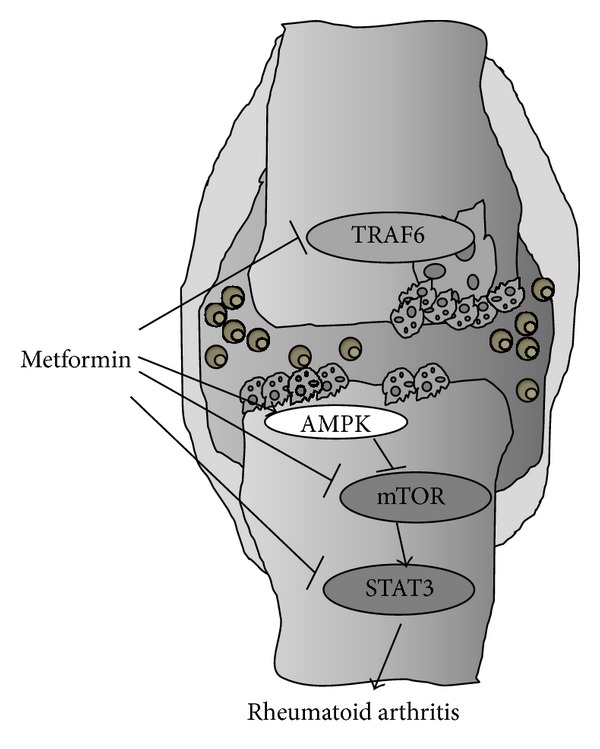
The signaling pathway that metformin uses to regulate T cell and osteoclasts in autoimmune arthritis.

To verify the observation that metformin reduced the expression of osteoclastogenic markers* ex vivo*, we investigated whether metformin could inhibit osteoclast differentiation* in vitro*. The results showed that metformin treatment inhibited osteoclast differentiation as determined by the TRAP staining assay (Figure 5(a)). We also measured osteoclastogenic markers mRNA expression levels in control osteoclasts and metformin-treated ones. The mRNA expression of the osteoclast-related markers, TRAP, cathepsin K, MMP-9, calcitonin receptor, carbonic II, and integrin 3*β*, decreased by meformin treatment. We also found that metformin suppressed the expression of HIF-1*α* mRNA whereas it induced expression of AMPK mRNA (Figure 5(b)). As the pharmacologic effect of metformin is largely dependent on AMPK, the activation of AMPK, mTOR, and STAT3 during osteoclastogenesis was addressed. Western blot analysis demonstrated that expressions of TRAF6,* p*mTOR, and* p*STAT3 (Y705 and S727) were reduced with metformin treatment. Also,* p*AMPK activation was increased (Figure 5(c)). Consistently, immunohistochemical staining of arthritic joints revealed that expressions of these molecules were significantly lower in metformin-treated CIA mice (Figure 5). Collectively, these results suggest that metformin suppresses osteoclastogenesis via inhibition of mTOR and STAT3, which is mediated by metformin-activated AMPK.

## 4. Discussion

In the present study, we demonstrated that metformin suppressed CIA via reciprocal regulation of Th17 cell and Treg cell. Metformin-activated AMPK seems to be involved in this regulation through the inhibition of mTOR and its downstream molecules, HIF-1*α* and STAT3.

As Th17 receives the spotlight in the pathogenesis of RA, a number of medications that target Th17 have been developed. IL-6 is essential cytokine for Th17 cell differentiation signaling through JAK-STAT pathway [15]. However, tocilizumab, an IL-6 receptor blocking antibody, is currently being used, whereas tofacitinib, a JAK inhibitor, is under clinical trials. The molecules shown to inhibit STAT3 or ROR*γ*T have demonstrated promising results in animal models.

Recently, metabolism has been raised as an important check point in regulating Th17/Treg balance [16]. Therefore, the role of AMPK, the master metabolic sensor, in T cell fate was investigated. Kang et al. reported that metformin-activated AMPK inhibited mTOR-STAT3 pathway contributing to a reduction in the population of Th17 cells [17]. Furthermore, our results revealed that metformin not only suppressed Th17 cells but also simultaneously enhanced Treg cells. This might be explained as the metabolic check point determines the differentiation of Th17 cell or Treg cell. Th17 cell and Treg cell are known to use different energy sources. For instance, Th17 cells are highly glycolytic while Treg cells have a high lipid oxidation rate [18]. The high glycolytic activity of Th17 cell is dependent on mTOR, which is inhibited by AMPK. mTOR enhances the glycolytic pathway promoting Th17 cell differentiation whereas AMPK inhibits mTOR and promotes mitochondrial oxidative metabolism to enhance Treg cell. Furthermore, the high glycolytic activity of Th17 cells is attributed to HIF-1*α*, a downstream molecule of mTOR pathway [19]. Th17 differentiation was impaired in HIF-1*α* deficient mice. In addition, HIF-1*α* promotes the nuclear translocation of ROR*γ*T and degrades Foxp3, resulting in a decrease in Treg cell [20]. Thus, AMPK regulate the balance between Th17 and Treg. AMPK has also been shown to negatively regulate the activation of STAT3 [21]. It is widely known that* p*STAT3 competes for the same binding locus as* p*STAT5 in IL-17 promoter to enhance IL-17 [22]. Therefore, activating AMPK and inhibiting mTOR are an efficient way to regulate Th17 and Treg concurrently. Our results revealed that metformin suppressed Th17 cell while increasing Treg cell in CIA mice. Though confocal microscopy showed an increase in STAT5 phosphorylation, it seems to result from reduced* p*STAT3 and consequently increase the number of Treg rather than the direct effect of metformin on STAT5 phosphorylation. Undeniably, metformin did not affect IL-2 induced STAT5 phosphorylation levels* in vitro* (data not shown).

It was reported that metformin ameliorated experimental autoimmune encephalitis [23] and collagen antibody induced arthritis [17], both involving Th17 cells. Our results confirmed that metformin-activated AMPK could suppress CIA via reciprocal regulation of Th17 cell and Treg cell. Therefore, future investigation of whether metformin can be used as a therapeutic in Th17-driven chronic inflammatory diseases is promising.

Metformin also suppressed osteoclastogenesis both* in vivo* and* in vitro*. The number of TRAP+ multinucleated cells was lower in the joints of metformin-treated mice. This may partially result from decreased expression of inflammatory cytokines that promote osteoclastogenesis via the upregulation of RANKL in the arthritic joint. Metformin stimulates osteoprotegerin and reduces RANKL expression in osteoblasts [24], and AMPK inhibits RANK signaling [25], which might also contribute to reduced osteoclastogenesis. To investigate the direct effect on osteoclastogenesis, preosteoclasts were cultured with M-CSF and RANKL in the presence or absence of metformin, decreasing osteoclastogenesis in the metformin-treated cells. Our results also revealed that the suppression was associated with an increase in AMPK phosphorylation and subsequent decrease in mTOR and STAT3 phosphorylation. The decreased expression of HIF-1*α* in metformin-treated osteoclasts verifies the recent observation that mTOR-HIF-1*α* pathway was critical in osteoclastogenesis [10].

To date, there is no prevention in the development of rheumatoid arthritis. However, proper early treatment can prevent progressive damage of the joints. In this study, the effect of metformin on joint destruction and regulation of immune cells in occurrence of rheumatoid arthritis was evaluated. Additionally, the prevention of rheumatoid arthritis by metformin should be further investigated.

Collectively, these results suggest a new role of metformin. Metformin has been shown to ameliorate autoimmune arthritis by regulating the Th17/Treg balance and inhibiting osteoclastogenesis through AMPK. In cases where secondary diabetes mellitus develops due to excessive corticosteroid use in RA patients, metformin can be the optimal therapeutic option due to its glucose lowering and anti-inflammatory properties.

## 5. Conclusions

The present study demonstrated that metformin had an anti-inflammatory effect on CIA due to the inhibition of Th17 cell differentiation and the upregulation of Treg cell differentiation along with the suppression of osteoclast differentiation. Our data suggest that metformin might be a potential candidate for therapeutic modulation of experimental animal model with rheumatoid arthritis.

## Figures and Tables

**Table 1:** Mouse primer sequences.

Name	Forward 5′→3′	Reverse 5′→3′
Interleukin(IL)-17 A	CCT CAA AGC TCA GCG TGT CC	GAG CTC ACT TTT GCG CCA AG
Aryl hydrocarbon receptor (Ahr)	AGC AGC TGT GTC AGA TGG TG	CTG AGC AGT CCC CTG TAA GC
Runt-related transcription factor 1 (RUNX1)	TAC CTG GGA TCC ATC ACC TC	GAC GGC AGA GTA GGG AAC TG
RAR-related orphan receptor gamma T (RORgT)	TGT CCT GGG CTA CCC TAC TG	GTG CAG GAG TAG GCC ACA TT
Forkhead box P3 (Foxp3)	GGC CCT TCT CCA GGA CAG A	GCT GAT CAT GGC TGG GTT GT
Tartrate resistant acid phosphatase (TRAP)	TCC TGG CTC AAA AAG CAG TT	ACA TAG CCC ACA CCG TTC TC
Integrin *β*3	CTG TGG GCT TTA AGG ACA GC	GAG GGT CGG TAA TCC TCC TC
Calcitonin receptor	CGG ACT TTG ACA CAG CAG AA	AGC AGC AAT CGA CAA GGA GT
Carbonic anhydrase II	TGG TTC ACT GGA ACA CCA AA	AGC AAG GGT CGA AGT TAG CA
Cathepsin K	CAG CAG AGG TGT GTA CTA TG	GCG TTG TTC TTA TTC CGA GC
Matrix metalloproteinases-9 (MMP-9)	CTG TCC AGA CCA AGG GTA CAG CCT	GAG GTA TAG TGG GAC ACA TAG TGG
HIF-la	AGG CCT AGA TGG CTT TGT GA	TAT CGA GGC TGT GTC GAC TG
AMPka	TGTTCCAGCA GATCCTTTCC	ATAATTGGGTGAGCCACAGC
*β*-Actin	GTA CGA CCA GAG GC A TAC AGG	GAT GAC GAT ATC GCT GCG CTG
